# Resource utilization by the Kori bustard in the Serengeti ecosystem

**DOI:** 10.1371/journal.pone.0221035

**Published:** 2019-09-04

**Authors:** Emmanuel Clamsen Mmassy, Roel May, Craig Jackson, Oddmund Kleven, Torgeir Nygård, Kjetil Bevanger, Eivin Røskaft

**Affiliations:** 1 Department of Biology, Norwegian University of Science and Technology, Trondheim, Norway; 2 Tanzania Wildlife Research Institute, Arusha, Tanzania; 3 Norwegian Institute for Nature Research, Trondheim, Norway; Ashoka Trust for Research in Ecology and the Environment, INDIA

## Abstract

This study aimed to understand the movement behaviour and utilization distributions of Kori bustards in space and time in the Serengeti ecosystem. A total of 14 individuals were tracked with the aid of GPS (Geographical positioning system) satellite transmitters, and their sexes were identified using DNA analysis. A species utilization distribution was estimated using the Brownian bridge movement model (hereafter dBBMM) in which the probability of being in an area is conditioned by starting and ending (GPS) relocations. Resource selections were analysed by comparing the GPS relocations with locations randomly placed within each individual’s region of utilization in a spatio-temporal approach. Vegetation information was derived from a Serengeti GIS vegetation map and Data Centre and was reclassified as Open grassland, Dense grassland, Shrubbed grassland, Treed grassland, Shrubland, and Woodland. The Shannon diversity index for vegetation was calculated based on the original vegetation classification. Used versus non-used habitats were contrasted using a generalized linear mixed-effects model with a binomial distribution. The results indicated that males were 21.5% more mobile than females, and movements were 6.3% more diffuse during the non-breeding period compared to the breeding period (7.59 versus 7.14, respectively). Contrasting models indicated that males preferred more open grasslands during the non-breeding period and also preferred closed and shrubbed grassland during the breeding period. Females preferred more woody vegetation during the non-breeding season compared to the breeding season. The most parsimonious model indicated that females preferred to stay closer to rivers and diverse areas during the non-breeding period whereas males preferred areas that were farther from rivers and homogenous. Homogeneous areas were preferred during the breeding period, and heterogeneous areas were preferred during the non-breeding period. We conclude that the movement behaviours of Kori bustards changes with the season and habitat. Further research is needed to understand the factors driving the seasonal movement of Kori bustards in the Serengeti ecosystem.

## Introduction

Habitat selection in animals helps maximize their fitness [1–4]. However, the behavioural process of habitat selection is not fixed and typically differs during periods of reproduction and between seasons [5]. Adjusting resource utilization in response to the spatial-temporal variation of food resources [6] allows animals to optimize their fitness, for example, through (partial) migration [7]. Differences in movement behaviour as induced by changing resources occur within individual populations and species, with some populations or individual species moving between habitats [7]. For conservation of the species, understanding their habitat use and distribution, and why a species may require different habitat types at different times during its life cycle and throughout the year, is important [8, 9].

Periods of reproduction may affect both the home range size and habitat selection. In birds, breeding pairs with chicks may have smaller home ranges because breeding birds require habitats with abundant food and safety for their young. Similarly, incubating females may have relatively small home ranges because their nests depend on incubation and protection [10]. However, there may be considerable variation in the way a habitat is used by different bird species, including when they are feeding, breeding, nesting or utilizing the resources to sustain chicks.

The Kori bustard is the heaviest flying bird that is indigenous to the grasslands and lightly wooded savannahs of southern and east Africa [11]; however, the habitat preferences of the two sexes (male and female) during different seasons with regard to vegetation diversity and distance to water are not clearly known. Kori bustard males differ from females by having a thick neck and black throat during the breeding season and a larger body size [12]. The Kori bustard is a polygynous species that shows elaborative courtship displays during the breeding season [13]. During the breeding season, the breeding males gather singly or in a dispersed display (loose lek-like) formation with white tail and neck feathers inflated to attract breeding females [12]. Females lay eggs on the small shallow scrapes near a small clump of grasses on the ground to hide from predators. The incubation period varies from three to four weeks, and the clutch size ranges from one to two eggs [13]. Similar to other bustards, the male Kori bustards do not participate in parental care, including incubation of the eggs and care of the young [12].

The distribution of these sub species has recently decreased due to various anthropogenic factors, including altered land use practices [14] and illegal harvests [15–19]. Data on the range and habitat utilization of this species within the Serengeti ecosystem are deficient, and thus, there is a need to obtain information that is currently lacking about the seasonal movements and habitats preferred by this species for conservation measures. *Ardeotis kori struthiunculus* is listed as near threatened by the IUCN and is included in Appendix II of the list of CITES species [20]. In general, the Kori bustard is regarded as a sedentary species, but local movements are largely thought to occur on foot in e.g., Kenya [21]. Knowledge about the movement behaviour of Kori bustards is limited, but species inhabiting semi-arid regions commonly undertake local movements in connection with rainfall events [22]. Compared to other bustard species, information on the subspecies *Ardeotis kori struthiunculus* is lacking. Therefore, this study will determine the habitat use and movements of the Kori bustard *Ardeotis kori struthiunculus* subspecies in the Serengeti ecosystem.

The protected Serengeti National Park and surrounding areas are home to a sizeable Kori bustard population. Given the continued decline of Kori populations elsewhere [17], this large protected area represents a potentially important conservation area. However, there is a lack of data on Kori bustard habitat utilization and ranging behaviour within the Serengeti ecosystem. To better understand the species’ habitat requirements and seasonal variations therein, we deployed satellite GPS units on Kori bustards in Serengeti National Park.

We hypothesized that: 1) the utilization distributions of male Kori bustards are larger than those of females during the breeding period because males move in different areas for courtship displays, 2) the mobility of female Kori bustards is smaller than males as they incubate and care for their young during the breeding period, and 3) the habitat preferences of male Kori bustards differ from those of females across seasons in response to spatial and temporal resource requirements.

## Methods

### Study area

**Fig 1:**
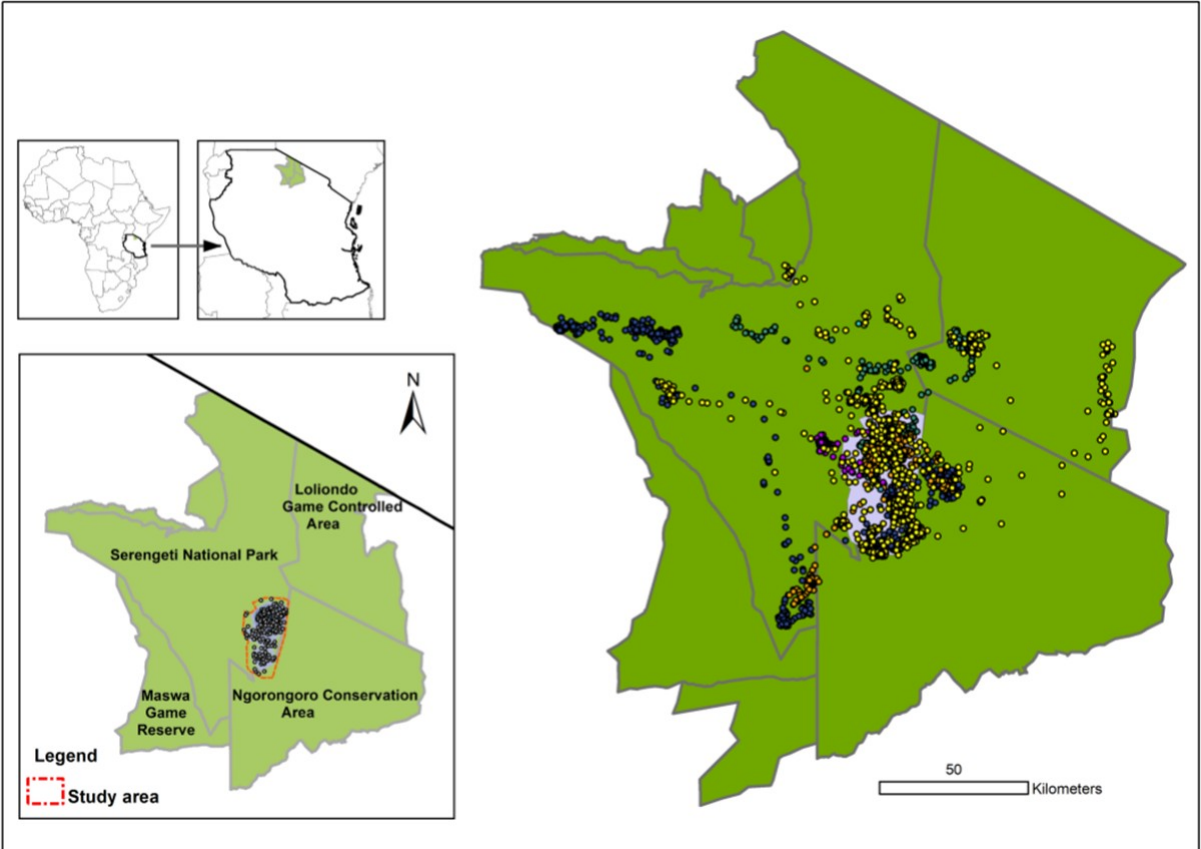
Map of the Serengeti-Mara ecosystem indicating the study area and movements of satellite GPS-collared Kori bustards.

The study was carried out in Serengeti National Park (SNP), occupying 14,763 km^2^. The Serengeti Volcanic Grasslands lie just south of the Tanzanian and Kenyan border and close to the equator (between latitudes 1° 28’-3° 17’ S and longitudes 33° 50’- 35° 20’ E). Climatically, the eco-region has a strong rainfall pattern within the seasonal tropics. The mean maximum temperatures are between 24° to 27°C, and the mean minimum temperatures are between 15° to 21°C. The mean annual rainfall varies from 1,050 mm in the northwest to 550 mm in the southeast [23]. The rainfall is strongly seasonal, with peaks between March and May (long rainfall) and between November and December (short rainfall) [24, 25]. SNP lies in the northern part of Tanzania (Fig 1). To the north, southeast, southwest, west, and northeast, SNP borders the Maasai Mara National Reserves in Kenya, the Ngorongoro Conservation Area, the Maswa Game Reserve, the Ikorongo and Grumeti Game Reserves and the Loliondo Game Controlled Area, respectively [26].

### Capture and tracking

This study was conducted in Serengeti National Park from 2013 to 2015. Kori bustards were located by driving slowly within the study area until an individual or a group of individuals were spotted by visual scanning and with the use of binoculars. A bird was approached by driving slowly at a speed of 20 km/hour and following the bird for approximately five minutes until it moved more slowly. Then, the vehicle stopped close to the bird, and a person jumped out and captured the bird by hand. We opted to use this capture method because a previously described method of capturing Kori bustards [27] and the Bengal florican bustard *Houbaropsis bengalensis* [28] using nets was not applicable in our study site, since the site is dominated mainly by grasses and a hard rocky substratum. This method [27] was recommended to capture our study species because of its effectiveness, and it also avoided injury to the Kori bustards compared to mist-nets [29]. After capture, the bird’s head was covered with a towel to prevent visual observation that might induce physiological stress, to calm the bird and to prevent injury to the bird handlers. Each transmitter was fitted in approximately less than ten minutes. No capture myopathy was observed, and the released birds walked away immediately.

Capture was conducted in the morning and during the pre-breeding season because during this period, birds increase their body weight due to intensive feeding in preparation for breeding. Following capture, morphometric measurements and a banding of tarsus using a coloured stainless-steel band were performed. Young fresh feathers were collected from captured Kori bustards for a molecular sex determination.

Eight birds were deployed with an AWT i.e. African Wildlife Tracking system, Model number: Satellite (Iridium) back-pack for Kori Bustard, Code: AWT-SKORIB, each weighing 210 grams, and six birds were deployed with ARGOS LC4, and 105 grams of GPS battery-powered transmitters that were attached on the back of 14 captured Kori bustards harnessed with Teflon ribbon material (South Africa Wildlife Tracking Company). Both telemetry devices were employed for birds early during the breeding season in February 2013 and 2014 to assess the seasonal movements and resource selections of both male and female Kori bustards during feeding and non-feeding periods (Table 1). Because the GPS schedules were set differently among individuals, we standardized the schedules by including only one daily relocation at 11:00 local time. All GPS devices included this hour in their schedule. This approach also reduced any temporal auto correlation in the data. All the included data represented 2943 positions for all 14 individuals (median 277 positions/individual; range = 25–350). The daily step length was on average 1087 m (95% CI: 177–6679 m).

**Table 1:** Overview of the radio-marked Kori bustards in SNP. Dates indicate the first (i.e., date caught) and last dates recorded by the GPS transmitters. The daily fixes indicate both the total number and the fixes during the breeding and non-breeding season, respectively.

ID	Sex	Weight	Tag type	Date from	Date to	Daily fixes
631	F	5.7	AWT	13/06/2013	10/09/2013	85 (17/68)
632	F	6.0	AWT	16/07/2013	02/09/2013	37 (0/37)
633	F	5.6	AWT	16/07/2013	22/08/2013	31 (0/31)
634	M	12.2	AWT	17/07/2013	15/08/2013	25 (0/25)
1279	F	4.8	AWT	23/02/2014	24/01/2015	327 (175/152)
1280	M	15.5	AWT	22/02/2014	17/12/2014	268 (139/129)
1281	M	14.8	AWT	21/02/2014	12/01/2015	286 (158/128)
1282	M	11.0	AWT	22/02/2014	12/07/2014	131 (119/12)
*1282	M	11.0	AWT	28/11/2014	14/02/2015	78 (76/2)
58963	F	5.4	ARGOS-MTI	05/03/2014	19/02/2015	343 (190/153)
67122	F	5.5	ARGOS-MTI	25/02/2014	19/02/2015	350 (197/153)
67123	F	4.5	ARGOS-MTI	04/03/2014	19/02/2015	345 (192/153)
83228	M	8.3	ARGOS-MTI	04/03/2014	19/02/2015	313 (191/122)
83229	F	4	ARGOS-MTI	04/03/2014	30/01/2015	324 (171/153)
95330	F	4.8	ARGOS-MTI	04/03/2014	Lost signal after three days	

### Data analysis

#### Molecular sex-determination

The sex of all Kori bustards was determined from collected feathers. The superior umbilicus and the tip (5–10 mm long) of the calamus of each feather were placed in a 2 mL Eppendorf tube, which contained 470 μL lysis buffer ATL (Qiagen, Hilden, Germany) and 30 μL Proteinase K (Qiagen). The feathers were digested overnight in an incubator at 56°C and pulse-vortexed twice during that period. Genomic DNA was extracted using the Maxwell 16 Research System (Promega, Madison, WI, USA) and the Maxwell 16 tissue DNA Purification Kit following the manufacturer’s protocol.

Sex was determined using the universal primers P2 and P8, which target the sex-linked chromobox-helicase-DNA-binding (CHD) genes CHD-W and CHD-Z [30]. The P8 primer was fluorescently (6FAM) labelled. A polymerase chain reaction (PCR) was performed with Qiagen’s Multiplex PCR Kit following the manufacturer’s protocol but used an 8.4 μL reaction volume. PCR products (1 μL) were mixed with a 0.14 μL GeneScan 500 LIZ (Applied Biosystems, Foster City, CA, USA) size standard and 6.16 μL Hi-Di formamide following capillary electrophoresis on an ABI 3130xl Genetic Analyzer (Applied Biosystems). Allele sizes were assigned using GeneMapper v5.0 software (Applied Biosystems). A single band (379 base-pairs) was amplified in males, and two bands (379 and 396 base-pairs) were amplified in females.

### Movement behaviour and utilization distributions in space and time

We estimated the utilization distributions for each individual, calendar year, and periods separately using the dynamic Brownian bridge movement model (hereafter, dBBMM) [31] with the brownian.bridge.dyn function in the movement library [32] of the statistical program R 3.2.2 [33]. We classified the two periodic seasons as breeding (December–June) and non-breeding (July–November). This methodology follows a Brownian bridge approach, which is a continuous-time stochastic model of movement in which the probability of being in an area is conditioned on starting and ending (GPS) relocations, the elapsed time between those relocations, and the mobility or speed of movement [34]. This approach is based on observed animal trajectories, incorporates temporal autocorrelations between consecutive relocations, and takes into account the measured biotelemetry error (set at 20 m). Additionally, dBBM allows changes in behaviour, using likelihood statistics to determine change points along the animal’s trajectory [31]. Only utilization distributions that were based on a minimum of 20 GPS relocations were included in the analyses. The diffusion coefficient of the underlying Brownian motion (${\hat{\sigma }}_{m}^{2}$), which is related to the mobility of the animal, was estimated per individual, calendar year and period. These settings rendered the best fit without the need to exclude animals from the analyses, which was assumed to be a proxy to evaluate how easily changes in behaviour could be identified [31]. The dBBMM gives an estimation of the probability of occurrence in an area based on an animal’s movement trajectories. We defined the spatial extent of space use by Kori bustards that are resident in the SNP as the 95% isopleth Brownian bridge utilization distribution boundary. We tested for the effects of the period and/or sex on both the (log-transformed) utilization distribution surface area and (log-transformed) Brownian motion variance using contrasting linear mixed effects models following an information theoretic approach while [35] controlling for the random effects of individuals nested within years. Models were constructed using the lmer function in the lme4 library [36]

### Resource selection in space and time

To analyse resource selection, we compared used GPS relocations with locations randomly placed within each individual’s region of utilization in a spatio-temporal approach (third-order selection) [37]. Individual regions of utilization were defined by the minimum convex polygon for each individual across seasons that the Kori bustards could have used. For a given individual, we created 10 random locations for each relocation (1:10 ratio), and randomly assigned temporal information of the used relocations to these points. After extracting the background information (vegetation, vegetation diversity and distance to rivers) for all the used and random locations, we obtained a dataset of the used locations with season and habitat associations and the random locations with the same season and their habitat associations. Vegetation information was derived from a vegetation map (Serengeti GIS and Data Centre) and reclassified into the following classes: Open grassland, Dense grassland, Shrubbed grassland, Treed grassland, Shrubland, and Woodland. The Shannon diversity index for vegetation was calculated within a 1-km circular moving window based on the original vegetation classification. The distance to rivers was calculated for each point and, thereafter, centred by the mean and scaled by their standard deviation. Thereafter, we compared used versus non-used/available types in contrasting resource selection functions using a generalized linear mixed-effects model with a binomial distribution, following a theoretical information approach [38]. Models were constructed using the glmer function in the lme4 library [39], and included the random effects for each individual that nested within a year.

First, we assessed the relative contribution of the three covariates in explaining the habitat preferences of Kori bustards. We also assessed pairwise interactions between covariates to investigate possible functional responses in habitat selection. Functional responses in habitat selection may occur when the preference for a feature depends on its relative availability [40], which in turn may depend on the spatial configuration of other features. Thereafter, we further assessed the potential effects of sex and seasonal periods on Kori bustard habitat selections. This assessment was performed by evaluating the goodness-of-fit of models, including each of the main habitat associations separately in interactions with the single, additive or first-order interactions, with sex and the seasonal period as factorial the covariates. We evaluated the predictive success of the most parsimonious resource selection function models, using *k*-fold cross-validation in employing an adjusted kxvlmer function [41] to take glmer models (kxvglmer). This evaluation was performed by training the model on a random sample of 80% of the data and testing the goodness-of-fit of the remaining 20% by using Spearman-rank correlations that were calculated between ten resource selection function bin ranks and area-adjusted frequencies for five ‘test-training’ sets.

All statistical modelling was performed using Theoretical Information approach.

#### Ethical note

Feather samples for DNA analysis were obtained from the Serengeti National Park under CITES permit no 29916 of 26/08/2016 with security stamp no. 1302816 issued by the Office of the Director of Wildlife, Dar es Salaam, Tanzania. Juvenile feathers were removed from each caught individual Kori bustard during collaring exercise for laboratory DNA analysis to identify individual sexes. As a rule, the radio transmitters applied to birds should not exceed 2–3% of their body mass [42]. Data were collected using the GPS satellite collars (AWT and ARGOS) weighed 210 and 105 grams, respectively, which were less than 2% of the Kori bustard’s body weight as recommended by ornithologists. The minimum weight of the collared individuals was 4 kg, whereas the maximum weight was 15.5 kg (Table 1).

The Tanzania Wildlife Research Institute Body of Directors through the Joint Management Committee (JMRC) and Research Program Committee (RPC), and the Tanzania Commission for Science and Technology (COSTECH) approved the Kori bustard research. The committees are composed of Tanzania Wildlife Management Authorities namely Tanzania National Parks, Ngorongoro Conservation Area Authority, Tanzania Forest Service and the Tanzania Wildlife Authority formally known as Wildlife Division. The permit to conduct wildlife research was granted by COSTECH whereas entries permit (TNP/HQ/C.10/13 dated 3rd July 2012) to Serengeti National Park was issued by the Tanzania National Parks. NB: Tanzania Wildlife Research Institute (TAWIRI) is the only Institute in Tanzania mandated to supervise all wildlife researches in Tanzania."

## Results

The 14 tracked Kori bustards included nine females and five males, as determined by the molecular sex analysis. There was no significant bias in sex regarding the number of daily fixes (Mann-Whitney U-test: W = 34, P = 0.228) or seasonal ratio of fixes (Mann-Whitney U-test: W = 15, P = 0.270). Overall, there was a tendency of higher mobility in males relative to females (F = 1.452, n = 14) as measured by the (log-transformed) Brownian motion variance. Nevertheless, the overall size of the annual utilization distributions did not differ between sexes (F = 0.032, n = 14) and was on average 411.4 km^2^ (SD interval: 91.3–1854.2). As some individuals were tracked (sufficiently) during one season only, annual utilization distributions that excluded these individuals encompassed on average 957.1 km^2^ (SD interval: 328.7–2785.8) and was still unaffected by sex (F = 0.07, n = 8).

**Fig 2:**
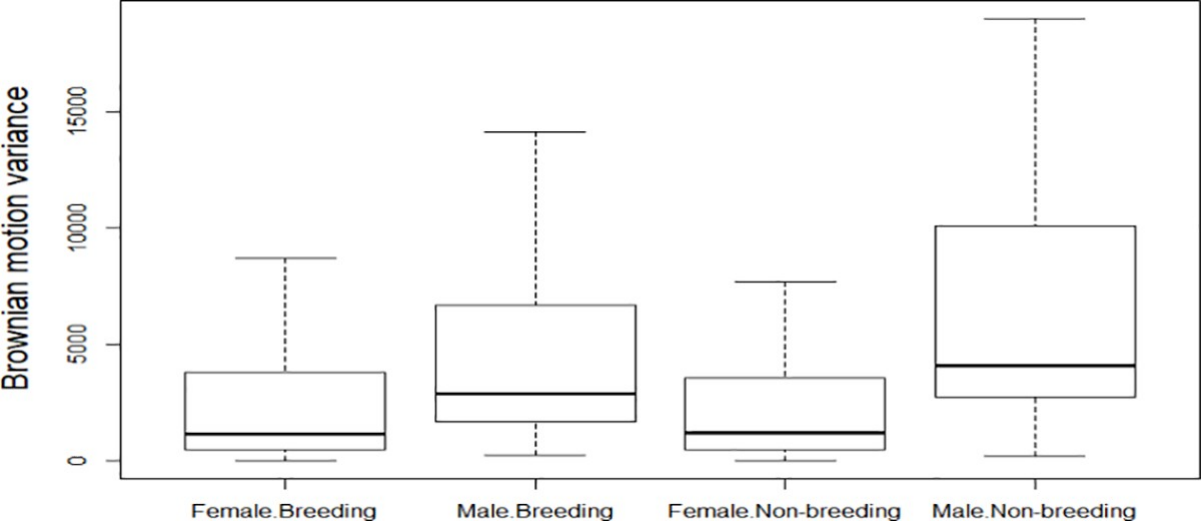
Mobility in Kori bustards in the Serengeti ecosystem as measured by the Brownian motion variance (measured in m^2^) by sex and seasonal period (breeding: December-June; non-breeding: July-November).

**Fig 3:**
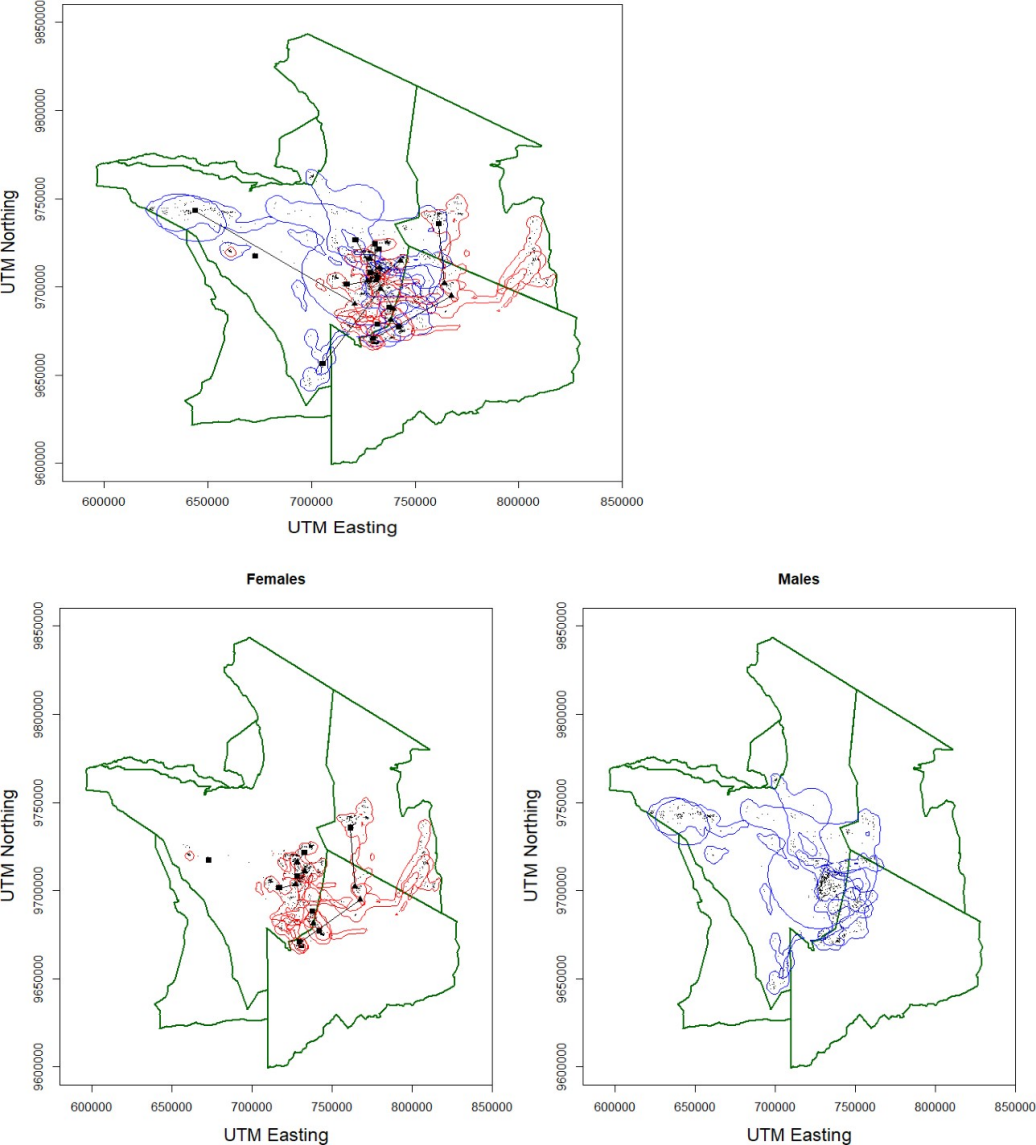
**Brownian bridge utilization distributions for male (blue) and female (red) Kori bustards in the Serengeti Ecosystem.** The points indicate the central location during the breeding (December-June; triangles) and non-breeding (July-November, squares) periods, interconnected by solid lines. The small dots represent the daily GPS locations.

When differentiating into seasonal movements, the (log-transformed) Brownian motion variance along the periodic animals’ track segments varied significantly by sex (F = 11.364, n = 2291) and seasonal period (F = 36.660, n = 2291) (Table 2, Fig 2). Males were 21.5% more mobile than females (8.27 m^2^ versus 6.81 m^2^, respectively), and males’ movements were 6.3% more diffuse during the non-breeding period compared to the breeding period (7.59 m^2^ versus 7.14 m^2^, respectively). We could not, however, identify any clear seasonal movements, even though some individuals did move longer distances (Fig 3, S1 Fig). Seasonal UD surface areas did not differ by sex and/or period (Table 2; mean UD: 327 km^2^, SD interval: 53–1997 km^2^).

**Table 2:** Akaike information criterion (AIC) results from the contrasting models assessing the effects of sex and/or seasonal period (breeding: December-June; non-breeding: July-November) on spatial use in Kori bustards in the Serengeti ecosystem. Models were constructed by assessing differentiations in mobility, as measured by the Brownian motion variance, (‘Mobility’ column) and the utilization distribution (UD) surface area (‘UD area’ column). The most parsimonious models are indicated in bold.

Model	df	Mobility	UD area
Intercept	4	10031	114.9
Period	5	9997	113.4
Sex	5	10026	114.7
Period + Sex	6	**9991**	113.6
Period*Sex	7	9992	**113.0**

The spatial configuration of the habitat available within the Kori bustard minimum convex polygons showed coinciding patterns. Woody vegetation was more likely to be found within more heterogeneous areas with a higher Shannon diversity (Kruskal-Wallis test: χ^2^ = 7863.1, df = 5, P < 0.001) and closer to rivers (χ^2^ = 2995.9, df = 5, P < 0.001). Habitats closer to rivers were also more likely to be more heterogeneous (Spearman rank correlation: ρ = -0.370, t = 68.251, P < 0.001).

**Fig 4:**
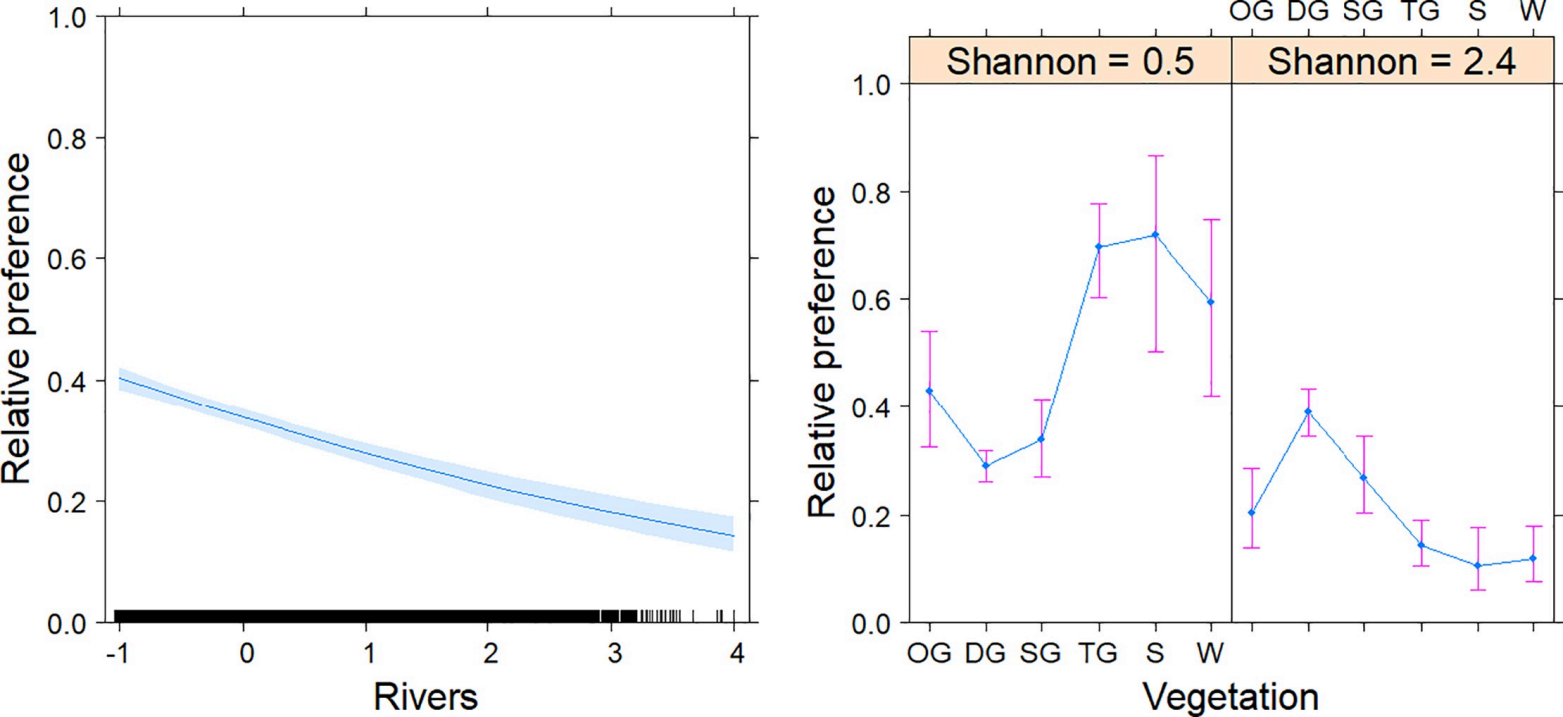
The most parsimonious model explaining habitat preferences in Kori bustards in the Serengeti Ecosystem. The model included the distance to rivers (centred and scaled) and a functional response of vegetation to Shannon diversity. The vegetation types are open grassland (OG), dense grassland (DG), shrubbed grassland (SG), treed grassland (TG), shrubland (S) and woodland (W). The relative preference indicates the relative probability of the back-transformed binomial scale excluding the intercept.

Of the three covariates, the Shannon diversity had no clear additive explanatory power on habitat selection in Kori bustards (F = 1.864). Conversely, vegetation (F = 5.147) and especially distance to rivers (F = 135.707) were important. Contrasting models with varying pair wise interactions (Table 3), used to assess the potential functional responses in habitat selection, indicated that Kori bustards varied their preference for vegetation depending on the diversity of their surroundings within a 1-km radius (Fig 4). This model was the most parsimonious model based on AIC values (Table 3) and had a strong predictive performance (cross-validation *r* = 0.988, *P*< 0.001). Kori bustards preferred to be in the vicinity of rivers. In more homogeneous areas, Kori bustards preferred more closed vegetation types.

**Table 3:** Modelling results of Kori bustard preferences for different types of vegetation, Shannon diversity and distance to rivers (centred and scaled), assessing various functional responses, within the Serengeti Ecosystem. The values indicate the effect sizes (F statistics) of the covariates in the models.

Covariates	Model 0	Model 1	Model 2	Model 3	Model 4	Model 5
*Vegetation*	5.147	5.139	5.589	4.601	4.849	4.8494
*Shannon [diversity]*	1.864		1.920	0.007	4.918	
*[Distance to] Rivers*	135.707	133.188	138.295	134.916	126.912	127.799
*Vegetation x Shannon*			19.322			
*Shannon x Rivers*				29.117		
*Vegetation x Rivers*					19.382	3.825
*df*	10	9	15	11	15	14
*AIC*	19537.59	19540.84	19452.49	19510.95	19528.02	19528.02
*ΔAIC*	85.10	88.35	**0.00**	58.46	75.53	75.53

**Fig 5:**
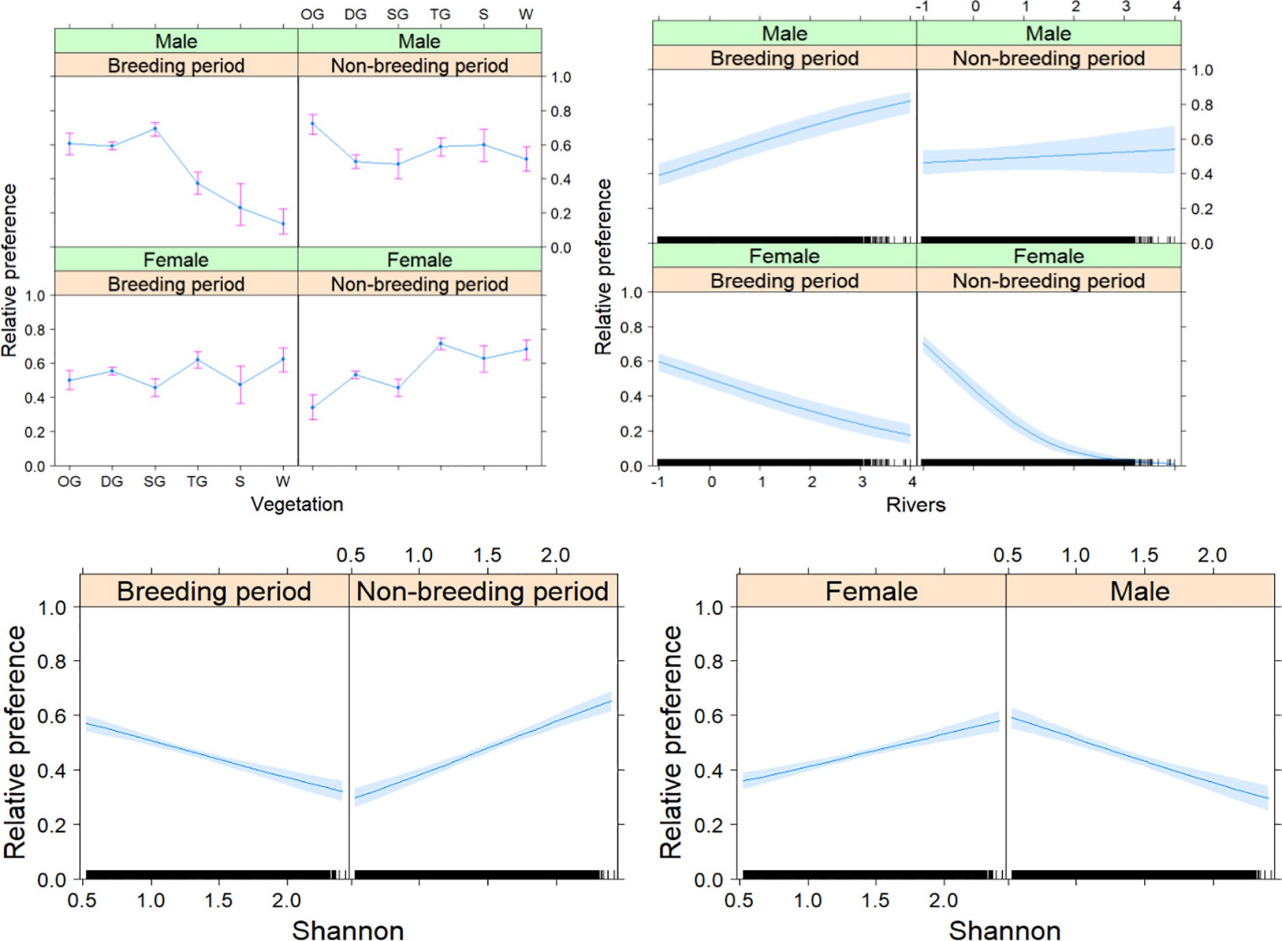
**The most parsimonious models explaining the sexual and seasonal differentiations in preferences for vegetation (upper left), distance to rivers (centred and scaled, upper right) and Shannon diversity (lower panels) for Kori bustards in the Serengeti Ecosystem.** Seasonal periods were categorized as breeding (December-June) and non-breeding (July-November) periods. The vegetation types are open grassland (OG), dense grassland (DG), shrubbed grassland (SG), treed grassland (TG), shrubland (S) and woodland (W). The relative preference indicates the relative probability on the back-transformed binomial scale excluding the intercept.

The most parsimonious model explaining the selection for vegetation indicated a sexual differentiation in the seasonal responses to habitat preference (Table 4, cross-validation *r* = 0.714, *P* = 0.088 [using seven bins due to clumpedness of the data]). Whereas males preferred more open grasslands, females preferred to use more woody vegetation (Fig 5). Males adjusted their preference according to the breeding versus the non-breeding period, with their highest preference for open grassland during the non-breeding period and increased preferences also for closed and shrubbed grassland during the breeding period. The female preference for woody vegetation types became less pronounced during the breeding season. The most parsimonious model explaining the selection for distance to rivers indicated a sexual differentiation in the seasonal responses to habitat preference (Table 4, cross-validation *r* = 0.952, *P*< 0.001). Females preferred to stay closer to rivers, even more so during the non-breeding period (Fig 5). Males preferred areas farther from rivers. The most parsimonious model explaining selections for vegetation diversity (Shannon diversity index) indicated differences between the sexes and seasonal responses to habitat preference (Table 4, cross-validation *r* = 0.818, *P* = 0.007). Females preferred more diverse areas contrary to males, who preferred more homogenous areas (Fig 5). During the breeding period, more homogeneous areas were preferred by Kori bustards, while an opposite preference was observed during the non-breeding period.

**Table 4:** Modelling results of Kori bustard preferences for different types of vegetation, Shannon diversity and distance to rivers (centred and scaled) by sex and breeding (December-June and non-breeding (July-November)) periods within the Serengeti Ecosystem. The most parsimonious models are indicated in bold.

Model	Vegetation			Shannon diversity			Distance to rivers		
	df	AIC	ΔAIC	df	AIC	ΔAIC	df	AIC	ΔAIC
.. x Sex x Period	**26**	**19352.05**	**0.00**	10	19413.58	1.93	**10**	**19104.04**	**0.00**
.. x (Sex + Period)	20	19404.13	52.08	**8**	**19411.65**	**0.00**	8	19114.75	10.71
.. x Sex	14	19486.45	134.40	6	19560.29	148.64	6	19219.27	115.22
.. *x Period	14	19576.73	224.68	6	19513.10	101.45	6	19418.68	314.64
Main effect (..)	8	19676.03	323.98	4	19691.69	280.04	4	19561.39	457.35
Intercept	3	19691.69	339.64	3	19692.54	280.89	3	19691.69	587.65

## Discussion

### Seasonal movements

In this study, we found sex-specific and seasonal differences in the movement behaviour of Kori bustards. Overall, there was a tendency of higher mobility in male Kori bustards; however, the overall size of the utilization distributions did not differ between sexes. Kori bustard movements varied significantly with sex and season. Males were more mobile than females, and males’ movements were more diffuse during non-breeding seasons compared to breeding seasons. Males had more movements than females because the parental responsibilities of Kori bustard males do not include incubation of the eggs or care for the young, which can be the cause of their high mobility. High mobility in males may also be caused by looking for females for courtship, as the breeding period was observed to be as long as nine months [43]. During the breeding period, males had less movement, as they may congregate in courtship display areas. The limited movement of females compared to males may be due to the critical nesting period of the Kori bustard in the studied ecosystem [43]. Breeding females were reported to have smaller movements, as they participate in the incubation of the eggs and parental duties [44, 45]. Different movement patterns of male and female Kori bustards in their habitats with respect to seasons can be explained by their differences in sexual activity [46].

However, we could not identify any clear seasonal movements, even though some individuals did move longer distances. Unclear movements (the absence of a well-pronounced movement) in different seasons may be due to the tendencies of some individuals to have strong movements, while these tendencies may be absent in other individuals. According to [43], Kori bustards in SNP have a relatively long breeding season of approximately nine months, which may be connected to the presence of small movements in some individuals during the non-breeding season, as they may breed in habitats with minimal favourable resources.

### Habitat preference

The spatial configuration of the habitats available within the Kori bustard minimum convex polygons and contrasting models based on AIC showed a species preference to woody vegetation, which is more heterogeneous and closer to rivers. The most parsimonious model explained the sexual differentiation in response to seasonal habitat/vegetation preferences wherein males preferred more open grasslands, and females preferred to use more woody vegetation. These preferences may reflect the species’ breeding system, where males and females require different microhabitats, as also observed in the Bengal florican *(Houbaropsis bengalensis)* [28]. Males require habitats that accelerate displays or conspicuousness to females (open grasslands), whereas females requires a habitat with woody vegetation that provides camouflage and food for their offspring [4, 43, 47, 48]. A study of the Little bustard (*Tetrax tetrax*) showed similar differences in habitat preferences between males and females [47]. Males selected open habitats without or with minimal vegetation cover for exposure, whereas females preferred habitats with a high vegetation cover for protection [47]. Males adjusted their preferences the most regarding the breeding versus the non-breeding period, with a highest preference for open grassland during the non-breeding period and an increased preference also for closed and shrubbed grassland during the breeding period. The female preference for woody vegetation types became less pronounced during the breeding season. It is possible that males moved to more open habitats for courtship displays prior to the onset of the main breeding season, and then, they returned to their habitat of preference [43, 49]. These two different habitats may provide different functions, including beneficial food resources in shrubbed grassland and displays for mating in open grassland, which influences periodical movements. However, more research is needed to quantify factors influencing habitat occupations by the Kori bustards in the Serengeti ecosystem. The female habitat preference positively correlated with habitat diversity, whereas males’ habitat preferences negatively correlated with habitat diversity; however, the factors influencing higher diversity and lower diversity preferences are not clear.

The most parsimonious model explaining the selection for distance to rivers indicated sexual differentiation in the seasonal responses to habitat preference (Table 4). Females preferred to stay closer to rivers, especially during the non-breeding period (Fig 5). This preference is reflected in the resource utilization, including water and food in the vicinity of rivers. Kori bustards have been shown to be locally nomadic in Namibia and sedentary in Zimbabwe and Botswana, where annual rainfall is more predictable [50]. This observation might be similar to our findings on Kori bustards’ preferences for the rivers in the Serengeti ecosystem. Males preferred areas farther from rivers. The most parsimonious model explaining the selection for vegetation diversity (Shannon diversity index) indicated differences between the sexes and seasonal responses to habitat preferences (Table 4). Females preferred more diverse areas contrary to males, who preferred more homogenous areas (Fig 5). During the breeding period more homogeneous areas were preferred, which switched to the opposite during the non-breeding period. This behaviour is because habitat heterogeneity/diversity offers more niches and dissimilar means of utilizing the environmental resources and thus provide species habitat preferences [51]. Thus, for the female Kori bustards, diverse habitats may be important in providing food, shelter from predators and shading for individuals and their young, as female Kori bustard participate in the incubation of the eggs and caring for the young. In heterogeneous habitats, different vegetation types have a considerable influence on the distributions and interactions of animal species [52], such as rats, reptiles and insects, which are the main composition of diet preferences of both young and adult individuals [53]. However, the use of homogeneous habitats during breeding might reflect the courtship displays of males to females in that displaying males can be easily seen prior to the onset of the main breeding season. After the courtship period, males return to their habitat of preference [43, 49]. Different movement patterns of male and female Kori bustards in their habitats with respect to seasons can be explained by their differences in sexual activity [46]. Based on Senyatso’s study in the South-African subspecies *Ardeotis kori kori*, female home ranges are confined by environmental conditions and the accessibility of resources [54].

### Conclusion and recommendation

Our study highlights how spatial use and habitat preferences shape the movements of Kori bustards in the Serengeti ecosystem. Overall, our results note the presence of distinct movement patterns related to breeding (December-June) and non-breeding (July-November) periods. We conclude that different movement patterns exist among individual Kori bustards during breeding and non-breeding periods, as females perform partial movements in their habitat of preference, whereas males migrate from one habitat to another. The Kori bustard habitat preferences in the Serengeti ecosystem are sex-specific and differ during different periods of the year, with males utilizing mostly shrubbed grassland and open grassland and females utilizing mostly woody vegetation with increasing habitat diversity. Finally, we recommend further studies on the factors that influence habitat preferences and differential movements of the Kori bustard in their ecosystem during different seasonal periods.

## Supporting information

S1 Fig Net-squared displacement (in 10^6^ km^2^) over Julian days from capture for six male (blue) and eight female (red) Kori bustards in the Serengeti Ecosystem during the breeding (grey top line, December-June) and non-breeding period (black top line, July-November).
(TIFF)

S1 Table Collar data of Kori bustard. (XLSX)
