# Supplementary figures and images for: Resource utilization by the Kori bustard in the Serengeti ecosystem
Source: PLoS One. 2019 Sep 4;14(9):e0221035. doi: 10.1371/journal.pone.0221035 (PMC6726138; doi:10.1371/journal.pone.0221035)

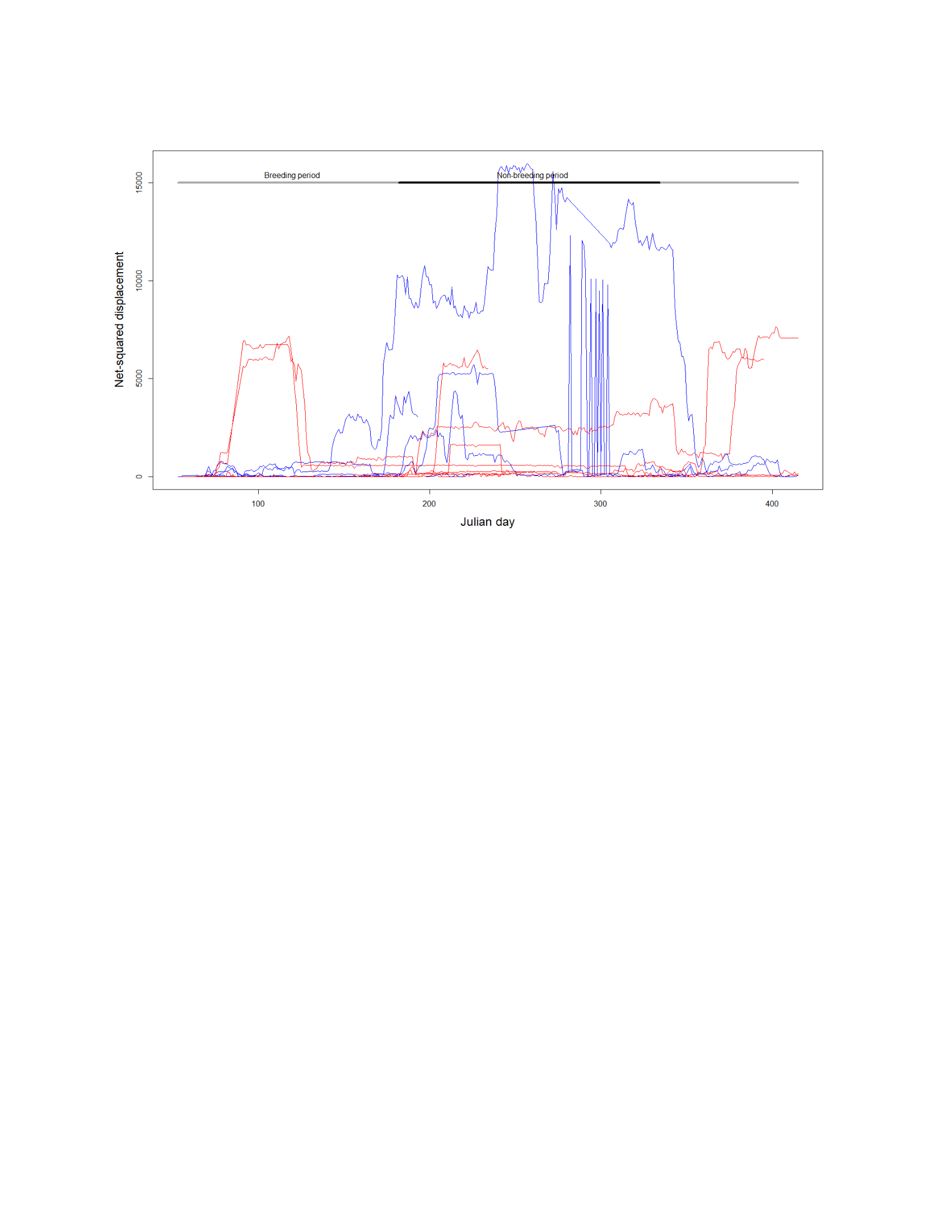

Supplement: S1 Fig — Net-squared displacement (in 106 km2) over Julian days from capture for six male (blue) and eight female (red) Kori bustards in the Serengeti Ecosystem during the breeding (grey top line, December-June) and non-breeding period (black top line, July-November). (TIFF) [file pone.0221035.s001.tiff]
